# On a Rare Disease of the Joints

**Published:** 1870-10

**Authors:** 


On a Rare Disease of the Joints.—Dr. Samuel Jackson, Emeritus Professor, etc., gives an account of several cases that have fallen under his observation, of a rare form of disease resembling rheumatoid arthritis, if they are not examples of this affection.

The peculiarity of the malady is, that it is confined to a single tissue—the ligamentous—the general health of the patient not being disturbed.

So far as his cases indicate the character of the disease, it seems to continue for years, to be incurable, but to have no direct tendency to cause death.

In one case in which the joint of the wrist and hand was the one affected, all the bones were dislocated. In another, both wrists were dislocated at every movement. It was evident that the cartilage had disappeared, and that the bones had become smooth by friction upon each other.

A third case had every joint dislocated and stiffened; yet the general health was good, and the patient lived for years with no pain save on an attempt at motion.

One patient traveled extensively, and received the best care and treatment for ten years. "During all this time the disease gradually progressed, stiffening the joints, until she became incapable of motion, and had to be carried about, fed like a child, and waited upon. And yet her general health is perfect, and has always been so; her organic functions are unimpaired, and her spirits are not affected."—*American Journal of Medical Science.*

